# Lipid parameters but not inflammatory indices predict hepatitis B vaccine responses in hemodialysis patients

**DOI:** 10.1093/ckj/sfag221

**Published:** 2026-07-01

**Authors:** Hilal Ekici, Arzu Mirza, Sevgi Baltacı, Davut Eren, Feyza İzci Çetinkaya, Mehmet Ceylan, Cansel Dağdelen, Sümeyye Yeşildağ, Sümeyra Koyuncu

**Affiliations:** Department of Infectious Diseases and Clinical Microbiology, Yozgat City Hospital, Yozgat, Türkiye; Ankara Provincial Directorate of Health, Public Health Services Presidency, Communicable Diseases Unit, Ankara, Türkiye; Department of Infectious Diseases and Clinical Microbiology, Sivas Numune Hospital, Sivas, Türkiye; Department of Nephrology, Kayseri City Hospital, Kayseri, Türkiye; Department of Infectious Diseases and Clinical Microbiology, Çankırı State Hospital, Çankırı, Türkiye; Department of Infectious Diseases and Clinical Microbiology, Bakırçay University Çiğli Training and Research Hospital, İzmir, Türkiye; Department of Infectious Diseases and Clinical Microbiology, Bakırçay University Çiğli Training and Research Hospital, İzmir, Türkiye; Department of Infectious Diseases and Clinical Microbiology, Şırnak İdil State Hospital, Şırnak, Türkiye; Department of Nephrology, Kayseri City Hospital, Kayseri, Türkiye

**Keywords:** hemodialysis, hepatitis B vaccine, high-density lipoprotein, immunometabolism, triglycerides

## Abstract

**Background:**

Hepatitis B virus (HBV) vaccination is essential in hemodialysis (HD) populations, yet seroprotection remains suboptimal. Inflammation-related hematologic indices are frequently used in clinical practice, but their ability to predict HBV vaccine response in HD patients is uncertain. We evaluated clinical and laboratory determinants of HBV vaccine response, with a focus on lipid-derived indices and inflammatory ratios.

**Methods:**

In this multicenter retrospective study, adult HD patients followed between 2015 and 2025 who received at least three doses of HBV vaccine and had available anti-HBs measurements were included (*n* = 341). Vaccine response was defined as anti-HBs ≥10 IU/L; non-response as <10 IU/L. Neutrophil-to-lymphocyte (NLR), platelet-to-lymphocyte (PLR), monocyte-to-lymphocyte (MLR), neutrophil percentage-to-albumin ratio (NPAR), and lipid ratios (TG/HDL, TC/HDL, LDL/HDL) were calculated. Additional analyses using percentile-based categorization of NPAR confirmed the absence of a significant association with vaccine response. Multivariable logistic regression and receiver operating characteristic analyses were performed.

**Results:**

After the primary vaccination series, seroprotection was 77.4% and increased to 88.6% after revaccination/boosters; it declined to 78.3% at 1 year and 69.2% at 2 years. Non-responders were older and had higher triglyceride levels and lower HDL cholesterol levels. In contrast, inflammatory indices (NLR, PLR, MLR, and NPAR) did not differ significantly between groups. These indices also demonstrated limited discriminative ability for predicting vaccine response. In multivariable analysis, HDL cholesterol remained positively associated with vaccine response [odds ratio (OR): 1.046, 95% confidence interval (CI): 1.006–1.086, *P* = .022], and triglyceride levels were negatively associated with vaccine response (OR: 0.992, 95% CI: 0.984–0.999, *P* = .024). The TG/HDL ratio showed borderline statistical significance (OR: 1.235, 95% CI: 0.983–1.551, *P* = .070), suggesting a potential but limited contribution to vaccine response prediction.

**Conclusions:**

In HD patients, hematologic inflammatory indices were not informative for HBV vaccine response, whereas an atherogenic lipid profile—particularly lower HDL and higher triglycerides—showed a consistent association. Lipid parameters may support risk stratification and tailored post-vaccination monitoring strategies.

## Graphical Abstract

**Graphical Abstract:**
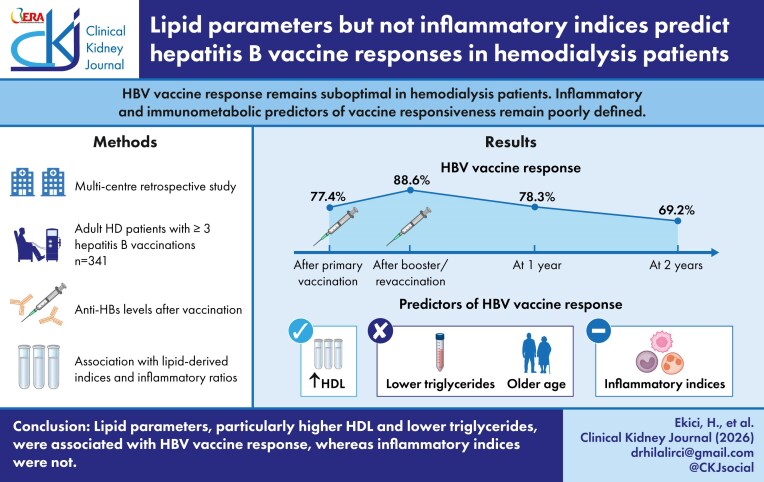

KEY LEARNING POINTS **What was known:**

- Hemodialysis patients have reduced and declining seroprotection following hepatitis B vaccination, highlighting the need for continued post-vaccination antibody monitoring.

**This study adds:**

- Conventional hematologic inflammatory indices (NLR, PLR, MLR, and NPAR) showed limited value in predicting hepatitis B vaccine response in this cohort.
- Lower HDL cholesterol and higher triglyceride levels were independently associated with reduced vaccine responsiveness, suggesting that immunometabolic factors may be more informative than routine inflammatory markers.

**Potential impact:**

- Lipid-related parameters may complement clinical risk stratification, although their predictive performance is modest and requires prospective validation before routine clinical use.

## INTRODUCTION

Hepatitis B virus (HBV) infection is an important cause of morbidity and mortality in patients with chronic kidney disease, particularly those receiving hemodialysis (HD). HD patients are at high risk for HBV infection because of frequent contact with blood and blood products, invasive procedures, and immune system impairment [1, 2]. Therefore, HBV vaccination is considered one of the core components of infection control in HD patients [3]. While hepatitis B vaccination can provide >90% seroprotection in the general population, this rate decreases substantially in HD patients, with response rates ranging from 50% to 80% across studies [4, 5].

Factors associated with inadequate vaccine response in HD patients include older age, male sex, diabetes mellitus, longer dialysis vintage, and malnutrition [6, 7]. Studies from Türkiye have also reported heterogeneous hepatitis B vaccine response rates and insufficient immunity in a substantial proportion of patients [8–10]. Serum albumin is an important biochemical marker reflecting nutritional status and inflammation in HD patients, and low albumin levels are associated with increased inflammation and impaired lymphocyte function [3, 11]. Previous studies have reported that low albumin levels may be associated with hepatitis B vaccine response; however, albumin alone may not adequately capture the complexity of inflammatory and metabolic processes [12, 13].

In recent years, hematological ratios reflecting systemic inflammation have attracted increasing attention in clinical practice. Indicators such as the neutrophil-to-lymphocyte ratio (NLR), platelet-to-lymphocyte ratio (PLR), and monocyte-to-lymphocyte ratio (MLR) have been associated with inflammation and prognosis in different populations [14, 15]. In addition to these indicators, the neutrophil percentage-to-albumin ratio (NPAR), derived by dividing the neutrophil percentage by the serum albumin level, has been defined as a novel index reflecting both inflammation and nutritional status; it has been shown to be associated with mortality and clinical outcomes in peritoneal dialysis and chronic kidney disease populations [16–18].

Meanwhile, the relationship between lipid metabolism and immune response has garnered increasing interest. It is well recognized that HDL cholesterol has anti-inflammatory and immunomodulatory properties, and that elevated triglycerides and an atherogenic lipid profile are linked to chronic inflammation [19, 20]. Although lipid-based ratios (e.g. TG/HDL, LDL/HDL) have been used to assess cardiovascular risk and inflammation, their association with hepatitis B vaccine response—particularly in HD patients—has been investigated only to a limited extent [21, 22].

The value of commonly used inflammatory indices for predicting hepatitis B vaccine response in HD patients remains uncertain, and the role of lipid-based immunometabolic markers in this context has not been adequately explored. In this study, we aimed to evaluate clinical and laboratory factors associated with antibody response after hepatitis B vaccination in HD patients, and to investigate—particularly focusing on NPAR—the potential roles of inflammation and lipid-based ratios in predicting hepatitis B vaccine response.

## MATERIALS AND METHODS

This multicenter retrospective study was designed to investigate clinical and laboratory factors affecting antibody response after hepatitis B vaccination in HD patients. The study was conducted in accordance with the STROBE (Strengthening the Reporting of Observational Studies in Epidemiology) guideline for observational research. Ethical approval was obtained from the Non-Interventional Clinical Research Ethics Committee. Because of the retrospective design, additional informed consent was not obtained, and all data were anonymized prior to analysis.

The study was conducted using data obtained between 2015 and 2025 from six centers. A total of 341 patients aged ≥18 years who were followed in HD programmes at these centers, had received at least three doses of hepatitis B vaccine, and had available follow-up anti-HBs results were included. Patients with missing baseline laboratory data, evidence of acute infection, comorbid conditions leading to hepatic failure, or those who had received only a single vaccine dose were excluded.

Data were extracted from hospital information management systems and archived records and recorded using a standardized data collection form. For each patient, age, sex, HD vintage, and comorbid conditions (diabetes mellitus, hypertension, coronary artery disease, pulmonary disease, chronic liver disease, and history of renal transplantation) were recorded. Laboratory parameters were recorded as the closest values obtained within 1 week prior to the first vaccine dose. Laboratory parameters included hematological measures such as hemoglobin, white blood cell count, neutrophil, lymphocyte, monocyte, and platelet counts, as well as serum albumin, total protein, urea, creatinine, uric acid, total cholesterol, HDL cholesterol, LDL cholesterol, and triglyceride levels. Blood samples were obtained as part of routine clinical practice and were generally based on pre-dialysis measurements. However, due to the retrospective and multicenter design, detailed information regarding fasting status and exact sampling time was not consistently available. Information on concomitant medications, including lipid-lowering agents (e.g. statins), diuretics, and immunosuppressive drugs, was also collected and incorporated into the multivariable analysis. All patients were screened for HIV/AIDS based on available medical records. Patients with active hepatitis B infection (HBsAg positivity) were not included in the study. A total of 20 patients were anti-HBc positive, indicating previous exposure to HBV; however, these patients were not excluded, as anti-HBc positivity does not represent active infection. All patients were receiving maintenance HD.

Hepatitis B vaccine response was assessed based on anti-HBs titers. Anti-HBs titers were measured using a chemiluminescent microparticle immunoassay (Architect i2000SR, Abbott Laboratories, USA). The upper limit of detection was 1000 IU/l. Following a vaccination series (single or double dose at months 0–1–2–6 or single or double dose at months 0–1–6), the anti-HBs measurement closest to 3 months after the last dose was considered the vaccine response and defined as the primary endpoint. All vaccines were recombinant HBsAg formulations with aluminum-based adjuvants. Standard dosing consisted of 20 µg per injection, whereas double-dose regimens involved 40 µg per administration. Patients with anti-HBs <10 IU/l were classified as ‘non-responders’, whereas those with anti-HBs ≥10 IU/l were classified as ‘responders’. Responders were further subdivided into ‘partial response’ (10–99 IU/l) and ‘complete response’ (≥100 IU/l). To jointly evaluate inflammation and nutritional status, several hematological and biochemical ratios were calculated.

NPAR was selected because it is readily available in routine clinical practice and reflects both inflammation and nutritional status. NPAR was calculated by dividing the neutrophil percentage by the serum albumin level (g/l). Albumin was measured in g/dl and converted to g/l for NPAR calculation (g/dl × 10). The distribution of NPAR was assessed using descriptive statistics and tested for normality using the Shapiro–Wilk test. Given the non-normal distribution (*P* < .001), non-parametric methods were applied. Patients were categorized into three groups based on data-driven cut-offs defined by the 25th and 75th percentiles of NPAR (<1.56, 1.56–2.11, >2.11). Comparisons between groups were performed using the Kruskal–Wallis test for continuous variables and chi-square test for categorical variables. Additionally, NLR (neutrophil/lymphocyte), PLR (platelet/lymphocyte), MLR (monocyte/lymphocyte), LDL/HDL, triglyceride/HDL, total cholesterol/HDL, and albumin/total cholesterol ratios were derived. Patients who achieved anti-HBs ≥10 IU/l after revaccination and booster doses were considered to have an overall vaccine response. Anti-HBs titers at year 1, year 2, and at the last follow-up date were also recorded. Due to the retrospective design, follow-up duration varied among patients, and not all individuals had complete longitudinal data. The primary outcome was defined as the anti-HBs response measured ∼3 months after completion of vaccination.

The association between vaccination schedules (0–1–6 single dose, 0–1–6 double dose, 0–1–2–6 double dose, and 0–1–2–6 single dose) and hepatitis B vaccine response was evaluated using the chi-square test. In all analyses, two-sided *P* < .05 was considered statistically significant.

Statistical analyses were performed using SPSS 20.0. Normality of continuous variables was assessed using the Kolmogorov–Smirnov test. Continuous variables are presented as mean ± standard deviation if normally distributed, or median (min–max) if not normally distributed. Categorical variables are presented as counts and percentages. Comparisons between responders and non-responders were performed using Student’s *t*-test for normally distributed variables and the Mann–Whitney *U* test for non-normally distributed variables, while categorical variables were compared using the chi-square test. For comparisons across three subgroups, ANOVA was used for normally distributed variables and the Kruskal–Wallis test for non-normally distributed variables. To identify factors associated with vaccine response, univariable and multivariable logistic regression analyses were performed; clinically relevant variables and those with *P* < .25 in univariable analysis were entered into the multivariable model. Backward stepwise selection was used in logistic regression. Additional multivariable models were constructed, including (i) age, HDL cholesterol, and triglycerides, and (ii) age and TG/HDL ratio, to assess potential collinearity. These analyses are presented in Supplementary Table S1. Model fit was evaluated using the Hosmer–Lemeshow test. ROC curves were generated to assess the discriminative performance of NPAR and other indices for vaccine response, and the area under the curve (AUC) was calculated. In all statistical analyses, *P* < .05 was considered statistically significant. Observations with missing data were excluded and complete-case analysis was performed.

## RESULTS

A total of 341 HD patients were included. Of these, 49.9% were women, and the median HD vintage was 36 months (6–288). The most common comorbidities were hypertension (82.4%), diabetes mellitus (38.1%), and coronary artery disease (33.1%). More than half of the patients (50.4%) received a double-dose 0–1–2–6-month vaccination schedule. All hepatitis B vaccines used in this study are recombinant HBsAg vaccines formulated with aluminum-based adjuvants; however, potential differences in immunogenicity between brands cannot be completely excluded. The serological response rate after the initial vaccination series was 77.4%, and the overall vaccine response rate increased to 88.6% after revaccination and booster doses. The seroprotection rate declined to 78.3% at the end of the first year and to 69.2% at the end of the second year (Table 1). No patients with a diagnosis of HIV/AIDS were identified in the study population.

**Table 1:** Demographic and clinical characteristics of the study population.

Variable	*n* (%) or Median (min–max)
Age (years)	64 (18–100)
Female sex	170 (49.9)
Male sex	171 (50.1)
Duration of hemodialysis (months)	36 (6–288)
Hypertension	281 (82.4)
Diabetes mellitus	130 (38.1)
Coronary artery disease	113 (33.1)
Pulmonary disease	35 (10.3)
Chronic liver disease	2 (0.6)
History of renal transplantation	24 (7.0)
Planned kidney transplantation	57 (16.7)
Current smoking	16 (4.7)
Vaccination schedule: 0–1–6 months, single dose	32 (9.4)
Vaccination schedule: 0–1–6 months, double dose	91 (26.7)
Vaccination schedule: 0–1–2–6 months, double dose	172 (50.4)
Vaccination schedule: 0–1–2–6 months, single dose	45 (13.5)
Anti-HBs ≥10 IU/l after primary vaccination series	264 (77.4)
Total anti-HBs ≥10 IU/l (including revaccination/booster doses)	302 (88.6)
Anti-HBs at 3 months (IU/l)	167 (0–1000)
Anti-HBs at last follow-up (IU/l)	130 (0–1000)^a^
Anti-HBs ≥10 IU/l at 1 year	267 (78.3)
Anti-HBs ≥10 IU/l at 2 years	236 (69.2)
Mortality	4 (1.2)

a Number of patients with available anti-HBs measurement at last follow-up: *n* = 331; upper measurement limit = 1000 IU/l.

Hematological parameters and inflammatory indices showed a wide distribution. When inflammatory ratios were evaluated, the median NLR, PLR, and NPAR were 3.12, 130.6, and 1.84, respectively, and the median serum albumin level was 3.7 g/dl (Table 2).

**Table 2:** Laboratory findings and inflammatory ratios.

Variable	Median (min–max)
White blood cell count (/µl)	7000 (2400–75 000)
Neutrophils (/µl)	4500 (1470–21 580)
Lymphocytes (/µl)	1500 (350–66 170)
Monocytes (/µl)	540 (30–2310)
Platelets (/µl)	204 000 (296–523 000)
NLR	3.12 (0.10–20.0)
MLR	2.78 (0.71–28.65)
PLR	130.6 (4.49–560.0)
NPAR	1.84 (0.39–7.22)
Creatinine (mg/dl)	6.7 (1.5–16.0)
Urea (mg/dl)	118 (24–265)
Uric acid (mg/dl)	6.1 (1.2–11.8)
Albumin (g/l)	37 (13–52)
Total protein (g/dl)	6.6 (4.2–8.4)
Total cholesterol (mg/dl)	163 (83–382)
Triglycerides (mg/dl)	149 (29–577)
HDL cholesterol (mg/dl)	38 (18–80)
LDL cholesterol (mg/dl)	102.5 (36–274)
Triglyceride/HDL ratio	3.89 (1.61–14.80)
Total cholesterol/HDL ratio	4.31 (4.10–9.55)
LDL/HDL ratio	2.58 (2.00–7.24)
Albumin/total cholesterol ratio	0.02 (0.01–0.86)

Abbreviations: HDL, high-density lipoprotein; LDL, low-density lipoprotein; TG, triglycerides; NLR, neutrophil-to-lymphocyte ratio; MLR, monocyte-to-lymphocyte ratio; PLR, platelet-to-lymphocyte ratio; NPAR, neutrophil percentage-to-albumin ratio.

When vaccination schedules were categorized into four groups (0–1–6 single dose, 0–1–6 double dose, 0–1–2–6 double dose, and 0–1–2–6 single dose), their distributions were 9.4%, 26.7%, 50.4%, and 13.5%, respectively. When vaccine response rates were evaluated according to vaccination schedules, no statistically significant difference was observed across groups (*P* = .079). The highest response rates were observed with the 0–1–2–6-month single-dose (87.0%) and double-dose (77.9%) schedules.

When responders (*n* = 264) and non-responders (*n* = 77) were compared, significant differences were observed in age, triglyceride levels, and HDL cholesterol levels. The non-responder group had higher age, higher triglyceride levels, and lower HDL cholesterol levels (*P* = .042, *P* = .015, and *P* = .036, respectively). In contrast, no significant differences were observed between groups for NPAR, NLR, PLR, or MLR. TG/HDL, total cholesterol/HDL, and LDL/HDL ratios were significantly higher in non-responders (*P* = .014, *P* = .016, and *P* = .031, respectively), whereas no difference was observed for the albumin/total cholesterol ratio (Table 3).

**Table 3:** Comparison of patients with and without hepatitis B vaccine response.

Variable	No response (anti-HBs <10) (*n* = 77)	Response (anti-HBs ≥10) (*n* = 264)	*P* value
Age (years)	68 (18–100)	62 (18–72)	**.042**
Female sex, *n* (%)	36 (46.8)	134 (50.8)	.219
Duration of hemodialysis (months)	36 (6–240)	36 (6–288)	.884
Hypertension, *n* (%)	64 (83.1)	217 (82.2)	.871
Diabetes mellitus, *n* (%)	34 (44.2)	96 (36.4)	.231
Coronary artery disease, *n* (%)	27 (35.1)	86 (32.6)	.690
Pulmonary disease, *n* (%)	10 (13.0)	25 (9.5)	.373
History of renal transplantation, *n* (%)	4 (5.2)	20 (7.6)	.472
White blood cell count (/µl)	7170 (5650–8540)	7000 (5860–8580)	.874
Neutrophils (/µl)	4700 (3600–5600)	4465 (3580–5825)	.965
Lymphocytes (/µl)	1400 (1000–1810)	1540 (1150–1960)	.132
Monocytes (/µl)	560 (400–700)	540 (420–700)	.982
Platelets (/µl)	201 000 (152 000–244 000)	204 000 (159 000–252 000)	.763
NLR	3.31 (2.33–4.54)	3.06 (2.15–4.29)	.309
PLR	130.38 (99.24–194.44)	131.06 (97.27–183.33)	.520
MLR	2.42 (1.92–3.63)	2.83 (2.12–3.78)	.138
NPAR	1.86 (1.56–2.05)	1.83 (1.57–2.12)	.741
Creatinine (mg/dl)	6.75 (5.02–8.55)	6.70 (5.10–8.40)	.743
Urea (mg/dl)	121 (87–143)	119 (90–147)	.651
Uric acid (mg/dl)	6.20 (5.50–7.00)	6.10 (5.20–7.10)	.593
Albumin (g/l)	37 (13–52)	37 (24–45)	.786
Total protein (g/dl)	6.6 (5.0–6.4)	6.6 (4.2–8.0)	.860
Triglycerides (mg/dl)	173 (127–235)	143 (100–211)	***.015***
HDL cholesterol (mg/dl)	35 (30–42)	40 (31–47)	***.036***
LDL cholesterol (mg/dl)	104 (84–130)	102 (76–127)	.492
TG/HDL ratio	4.63 (3.19–7.53)	3.67 (2.38–5.95)	.***014***
Total cholesterol/HDL ratio	4.67 (3.84–5.55)	4.19 (3.41–5.30)	***.016***
LDL/HDL ratio	2.83 (2.17–3.63)	2.46 (1.82–3.38)	***.031***
Albumin/total cholesterol ratio	0.02 (0.01–0.02)	0.02 (0.01–0.02)	.466

CAD, coronary artery disease; HDL, high-density lipoprotein; LDL, low-density lipoprotein; TG, triglycerides; NLR, neutrophil-to-lymphocyte ratio; MLR, monocyte-to-lymphocyte ratio; PLR, platelet-to-lymphocyte ratio; NPAR, neutrophil percentage-to-albumin ratio. Bold values indicate statistical significance (P < 0.05)

NPAR values demonstrated a non-normal distribution (Shapiro–Wilk *P* < .001). Patients were stratified into three groups based on percentile-derived cut-offs (<1.56, 1.56–2.11, and >2.11). No significant association was observed between NPAR categories and vaccine response (Kruskal–Wallis χ² = 0.43, *P* = .807; chi-square *P* = .806). Significant differences were identified across NPAR groups for several clinical and laboratory parameters. Higher NPAR levels were associated with older age (*P* = .013), increased neutrophil counts, decreased lymphocyte counts, and higher NLR (all *P* < .001). Additionally, serum albumin, total protein, and the albumin-to-total cholesterol ratio decreased significantly with increasing NPAR (all *P* < .001). In contrast, classical lipid parameters and renal function markers did not differ significantly between groups (all *P* > .05) Table 4. Overall, higher NPAR levels were associated with increased systemic inflammation and poorer nutritional status.

**Table 4:** Comparison of clinical and laboratory parameters according to NPAR categories.

Variable	NPAR <1.56 (*n* = 81)	1.56–2.11 (*n* = 175)	>2.11 (*n* = 85)	*P* value
Age (years)	58 (47–69)	66 (53–75)	64 (51–73)	.013^a^
Neutrophils (/µl)	3560 (3120–4200)	4720 (3810–5750)	5600 (4400–7300)	<.001^a^
Lymphocytes (/µl)	1900 (1580–2400)	1400 (1105–1935)	1310 (1000–1800)	<.001^a^
NLR	2.13 (1.50–3.20)	3.44 (2.20–4.80)	4.75 (3.10–6.90)	<.001^a^
Albumin (g/l)	40 (37–43)	37 (34–40)	34 (31–38)	<.001^a^
Total protein (g/dl)	6.9 (6.5–7.4)	6.6 (6.2–7.0)	6.3 (5.9–6.8)	<.001^a^
Albumin/Total cholesterol ratio	0.024 (0.020–0.028)	0.022 (0.018–0.025)	0.019 (0.016–0.022)	<.001^a^

a Kruskal–Wallis test [continuous variables, median (IQR)] NPAR categories were defined based on the 25th and 75th percentiles of the study population (<1.56, 1.56–2.11, and >2.11).

When groups defined by anti-HBs levels -no response (<10 IU/l), partial response (10–99 IU/l), and complete response (≥100 IU/l)- were compared, statistically significant differences were observed in total cholesterol (*P* = .049) and LDL cholesterol (*P* = .049) levels. No significant differences were observed among groups for triglyceride and HDL cholesterol levels, inflammatory ratios, or serum albumin levels (*P* > .05).

When lipid ratios were evaluated according to antibody response levels, the TG/HDL ratio differed significantly across groups (*P* = .034). The TG/HDL ratio was higher in the non-responder group, with a stepwise decreasing trend toward the partial and complete response groups. This finding suggests an inverse relationship between an atherogenic lipid profile and anti-HBs response levels.

Total cholesterol/HDL and LDL/HDL ratios were also higher in the no-response group, but these differences were at the borderline of statistical significance (*P* = .052 and *P* = .057, respectively). In contrast, the albumin/total cholesterol ratio did not differ significantly by antibody response level. Concomitant use of lipid-lowering therapy (*P* = .962), statins (*P* = .969), and diuretics (*P* = .812) was not significantly associated with vaccine response. Immunosuppressive therapy showed a borderline association (OR: 3.29, *P* = .057), but was not retained in the final model. Concomitant medications, including lipid-lowering therapy, statins, diuretics, and immunosuppressive agents, were also evaluated, and the results are presented in Supplementary Table S2.

In ROC analyses, none of NPAR, NLR, MLR, PLR, or the LDL/HDL ratio demonstrated a significant ability to discriminate HBV vaccine response (AUC = 0.488, *P* = .741; AUC = 0.538, *P* = .309; AUC = 0.445, *P* = .138; AUC = 0.524, *P* = .520; and AUC = 0.569, *P* = .066, respectively). Lymphocyte count did not differ significantly between responders and non-responders (*P* = .132), and the calculated effect size was negligible (*r* = 0.08). In multivariable logistic regression analysis, HDL cholesterol showed a positive association with vaccine response, whereas triglyceride levels showed a negative association. TG/HDL ratio demonstrated borderline significance, NPAR and other inflammatory indices were eliminated from the model (Table 5). Concomitant use of lipid-lowering therapy (OR: 1.06, 95% CI: 0.09–12.32, *P* = .962), statins (OR: 1.05, 95% CI: 0.08–13.02, *P* = .969), and diuretics (OR: 1.08, 95% CI: 0.56–2.09, *P* = .812) remained non-significant in the multivariable model. Immunosuppressive therapy was also evaluated in multivariable analysis but was not retained in the final model due to lack of statistical significance.

**Table 5:** Multivariable logistic regression analysis for predictors of HBV vaccine response.

Variable	OR	95% CI	*P* value
Age (years)	0.984	0.966–1.002	.079
HDL cholesterol (mg/dl)	1.046	1.006–1.086	**.022**
Triglycerides (mg/dl)	0.992	0.984–0.999	**.024**
TG/HDL ratio	1.235	0.983–1.551	.070

HDL, high-density lipoprotein; TG, triglycerides; CI, confidence interval.

Variable selection was performed using the backward stepwise method.

Model fit was assessed using the Hosmer–Lemeshow goodness-of-fit test.

Model discrimination: AUC = 0.576 (95% CI: 0.505–0.647).Bold values indicate statistical significance (P < 0.05).

ROC analysis based on predicted probabilities derived from the final multivariable logistic regression model demonstrated modest discriminatory performance, with an AUC of 0.576 (95% CI 0.505–0.647, *P* = .042). Hosmer–Lemeshow testing suggested acceptable model calibration.

## DISCUSSION

HD patients exhibit reduced seroconversion rates; therefore, intensified vaccination strategies such as higher antigen doses and additional doses are widely recommended.

In this multicenter, retrospective study of 341 HD patients, the serological response rate (anti-HBs ≥10 IU/l) after a primary hepatitis B vaccination series was 77.4%, and seroprotection increased to 88.6% after revaccination and booster doses. During follow-up, this rate declined to 78.3% in the first year and 69.2% in the second year. These findings are consistent with previous reports indicating that HBV vaccine response is lower in the HD population than in the general population and that antibody titers may decline over time [7, 12]. Even when high initial seroprotection rates are achieved, these results once again emphasize the clinical importance of regular anti-HBs monitoring and appropriate booster strategies in HD patients. Indeed, CDC/ACIP guidance recommends continued serological monitoring as long as risk persists in dialysis patients and administration of additional doses when necessary [23].

In our study, older age in the non-responder group is consistent with the literature [13, 24]. The adverse effects of immunosenescence on antigen presentation, T-cell help, and B-cell function may contribute to a weaker vaccine response [24]. However, the borderline significance of age in multivariable analysis suggests that the effect of age may be shared with other biological axes, such as lipid profile, and with unmeasured confounders [24]. When considered alongside classic evidence showing that vaccination at earlier stages of chronic kidney disease yields a stronger serological response, this finding supports the importance of an ‘early vaccination’ approach [25].

The linear trend observed between vaccination schedule and vaccine response suggests that ‘dose intensity and timing’ in HBV immunization may influence vaccine response. Because inadequate immune response is common in chronic kidney disease and dialysis populations, intensified schedules—such as shorter intervals or four-dose protocols—have been reported to yield higher antibody responses [26]. For example, HBV vaccination schedules administered over a shorter period with three or four doses have shown faster or similar response rates compared with standard 0–1–2–6-month regimens, indicating that vaccine response may vary according to ‘schedule characteristics’. This supports the notion that a more immunogenic vaccination approach may contribute to improved response in HD patients [27].

One of the notable findings of this study is that NLR, PLR, MLR, and particularly NPAR were insufficient for predicting HBV vaccine response. Given that these indices have previously been associated with mortality and clinical outcomes in chronic kidney disease and dialysis populations, it was necessary to examine whether they also reflect a specific immune outcome such as HBV vaccine response. This result does not imply that inflammatory indices are universally uninformative; rather, it suggests that they may be limited in capturing HBV vaccine–specific humoral immunity. Ratios such as NLR, PLR, and MLR are relatively crude markers of systemic inflammation and may not directly reflect more specific immune processes critical for vaccine response, including memory B-cell function, T-follicular helper responses, and germinal center activity [3, 19]. In this study, the relationship between inflammatory indices (NLR, PLR, MLR, and NPAR) and HBV vaccine response was evaluated in detail. The findings indicate that these indices have limited discriminative ability for predicting HBV vaccine response.

In our study, inflammatory indices such as NLR, PLR, MLR, and NPAR did not demonstrate significant predictive value for vaccine response. While these markers have been widely associated with overall mortality and systemic inflammation, their limited performance in predicting hepatitis B vaccine response may be explained by underlying immunological mechanisms. These indices primarily reflect non-specific innate immune activation, whereas vaccine-induced immunity is predominantly driven by adaptive immune responses, including T-cell–dependent B-cell activation and antibody production. Therefore, these indices may not adequately capture antigen-specific humoral immunity, which could explain their limited discriminative ability in this cohort [3, 16–18].

The ‘ceiling effect’ of chronic low-grade inflammation common in the HD population may have reduced the discriminative capacity of hematological ratios measured at a single time point [7]. In addition, the retrospective design, heterogeneity in measurement timing, and inter-center differences may have weakened the statistical signal of these indices.

In the present study, NPAR demonstrated a non-normal distribution and was therefore evaluated using percentile-based categorization. Although higher NPAR levels were strongly associated with multiple hematological parameters, including increased neutrophil counts, decreased lymphocyte counts, and elevated NLR, as well as lower albumin and total protein levels, these findings primarily reflect systemic inflammation and nutritional status rather than disease-specific immune responses.

Importantly, despite these biologically consistent associations, no significant relationship was observed between NPAR and HBV vaccine response. This suggests that systemic inflammatory markers, although relevant for overall clinical status, may not adequately capture antigen-specific humoral immunity. HBV vaccine response is a complex process involving coordinated T-cell–dependent B-cell activation and antibody production, mechanisms that are not directly reflected by simple hematological ratios such as NPAR.

Taken together, our findings indicate that while NPAR is a useful marker of inflammation and nutritional status in HD patients, its utility in predicting vaccine-specific immune response appears limited. These results highlight the need for more specific and mechanistically relevant biomarkers to better understand and predict vaccine responsiveness in this population [3, 19].

In our study, the most consistent and clinically meaningful associations were observed between lipid parameters and HBV vaccine response. Higher triglyceride levels, lower HDL cholesterol levels, and significantly increased TG/HDL, total cholesterol/HDL, and LDL/HDL ratios in non-responders are notable.

Although HDL cholesterol and triglyceride levels showed directional associations in multivariable analysis, their modest effect sizes and borderline statistical significance suggest that these parameters alone may not be sufficient to reliably predict HBV vaccine response. This interpretation is supported by the limited discriminative performance of the model in ROC analysis.

Similarly, large population-based studies in recent years have reported that low HDL cholesterol and high triglyceride levels are associated with weakened antibody responses following hepatitis B vaccination and that an atherogenic lipid profile may adversely affect humoral immunity to vaccination [1, 2].

HDL cholesterol is not only a metabolic marker but also an active regulator of immune responses. It modulates antigen-presenting cell function, cytokine production, and T-cell activation, all of which are essential for effective vaccine-induced immunity. Therefore, the positive association between HDL levels and vaccine response observed in our study may reflect both metabolic and immunological mechanisms [20].

The anti-inflammatory and immunomodulatory properties of HDL—including endotoxin-binding capacity and regulatory effects on antigen presentation—are well described in the literature [20]. In contrast, hypertriglyceridemia and an atherogenic lipid profile are associated with chronic inflammation and immune dysfunction, which may negatively affect antibody maturation processes required for vaccine response [2, 21, 22]. In this context, our findings suggest that an immunometabolic profile may play a more important role in explaining HBV vaccine response in HD patients than classical inflammatory ratios [1, 20]. Nevertheless, the limited AUC values of lipid ratios in ROC analyses indicate that these parameters may be better considered as complementary components within multivariable risk models rather than as stand-alone screening tests. Although the final model demonstrated statistically significant discriminatory ability, its predictive performance remained modest. Therefore, these findings should be interpreted as exploratory and should not be considered sufficient for stand-alone clinical prediction.

This study has several limitations. First, its retrospective multicenter design may have introduced residual confounding and inter-center variability. Second, inflammatory and biochemical parameters were evaluated at a single time point, and the timing of measurements relative to vaccination was not fully standardized. Third, unmeasured factors potentially affecting vaccine response—including nutritional status, dialysis adequacy, and cellular immune function—could not be comprehensively assessed. Finally, although the final multivariable model demonstrated statistically significant discriminatory ability, its overall predictive performance remained modest.

## CONCLUSIONS

In this multicenter retrospective study of HD patients, classical hematological inflammatory indices, including NLR, PLR, MLR, and NPAR, demonstrated limited value in predicting HBV vaccine response. In contrast, lipid-related parameters—particularly lower HDL cholesterol and higher triglyceride levels—showed more consistent associations with vaccine responsiveness. These findings suggest that immunometabolic profile may contribute more substantially than routine inflammatory ratios to the heterogeneity of HBV vaccine response in HD patients. Although the discriminatory performance of the final model remained modest, the results support further prospective studies integrating metabolic and immune biomarkers to improve individualized vaccination strategies and post-vaccination monitoring in this high-risk population.

## Supplementary Material

sfag221_Supplemental_Files

## Data Availability

The data underlying this study are not publicly available because they contain potentially identifiable patient information. De-identified data are available from the corresponding author upon reasonable request and subject to approval by the participating institutions and the relevant ethics committee.
