# Supplementary material for: Lipid parameters but not inflammatory indices predict hepatitis B vaccine responses in hemodialysis patients
Source: Clin Kidney J. 2026 Jul 1;19(8):sfag221. doi: 10.1093/ckj/sfag221 (PMC13437067; doi:10.1093/ckj/sfag221)
Supplement: Supplementary file 1 [file sfag221_supplemental_files.zip › 131 Supplementary Tables.docx]

**Supplementary Table S1. Multivariable Logistic Regression Models Evaluating Predictors of HBV Vaccine Response**

Model 1: Age + HDL + Triglycerides

| **Variable** | **OR** | **95% CI** | **p-value** |
| --- | --- | --- | --- |
| Age | 0.965 | 0.932 – 0.998 | 0.041 |
| HDL cholesterol | 1.022 | 0.998 – 1.047 | 0.071 |
| Triglycerides | 0.998 | 0.995 – 1.001 | 0.091 |

Model 2: Age + TG/HDL Ratio

| **Variable** | **OR** | **95% CI** | **p-value** |
| --- | --- | --- | --- |
| Age | 0.967 | 0.935 – 1.000 | 0.052 |
| TG/HDL ratio | 1.235 | 0.983 – 1.551 | 0.070 |

**Supplementary Table S2. Concomitant medications and HBV vaccine response**

| **Variable** | **No response n/N (%)** | **Response n/N (%)** | **p-value** |
| --- | --- | --- | --- |
| Lipid-lowering therapy | 17/72 (23.6) | 55/72 (76.4) | 0.814 |
| Statin use | 16/68 (23.5) | 52/68 (76.5) | 0.834 |
| Fibrate use | 2/8 (25.0) | 6/8 (75.0) | 0.868 |
| Diuretic use | 19/80 (23.8) | 61/80 (76.3) | 0.775 |
| History of renal transplantation | 4/24 (16.7) | 20/24 (83.3) | 0.472 |
| Prednisone use | 6/25 (24.0) | 19/25 (76.0) | 0.860 |

Values are presented as n/N (%). Comparisons were performed using chi-square or Fisher’s exact test, as appropriate.
