# Supplementary material for: Lipid parameters but not inflammatory indices predict hepatitis B vaccine responses in hemodialysis patients
Source: Clin Kidney J. 2026 Jul 1;19(8):sfag221. doi: 10.1093/ckj/sfag221 (PMC13437067; doi:10.1093/ckj/sfag221)
Supplement: Supplementary file 1 [file sfag221_supplemental_files.zip › ROC_curve_final_model.pdf]

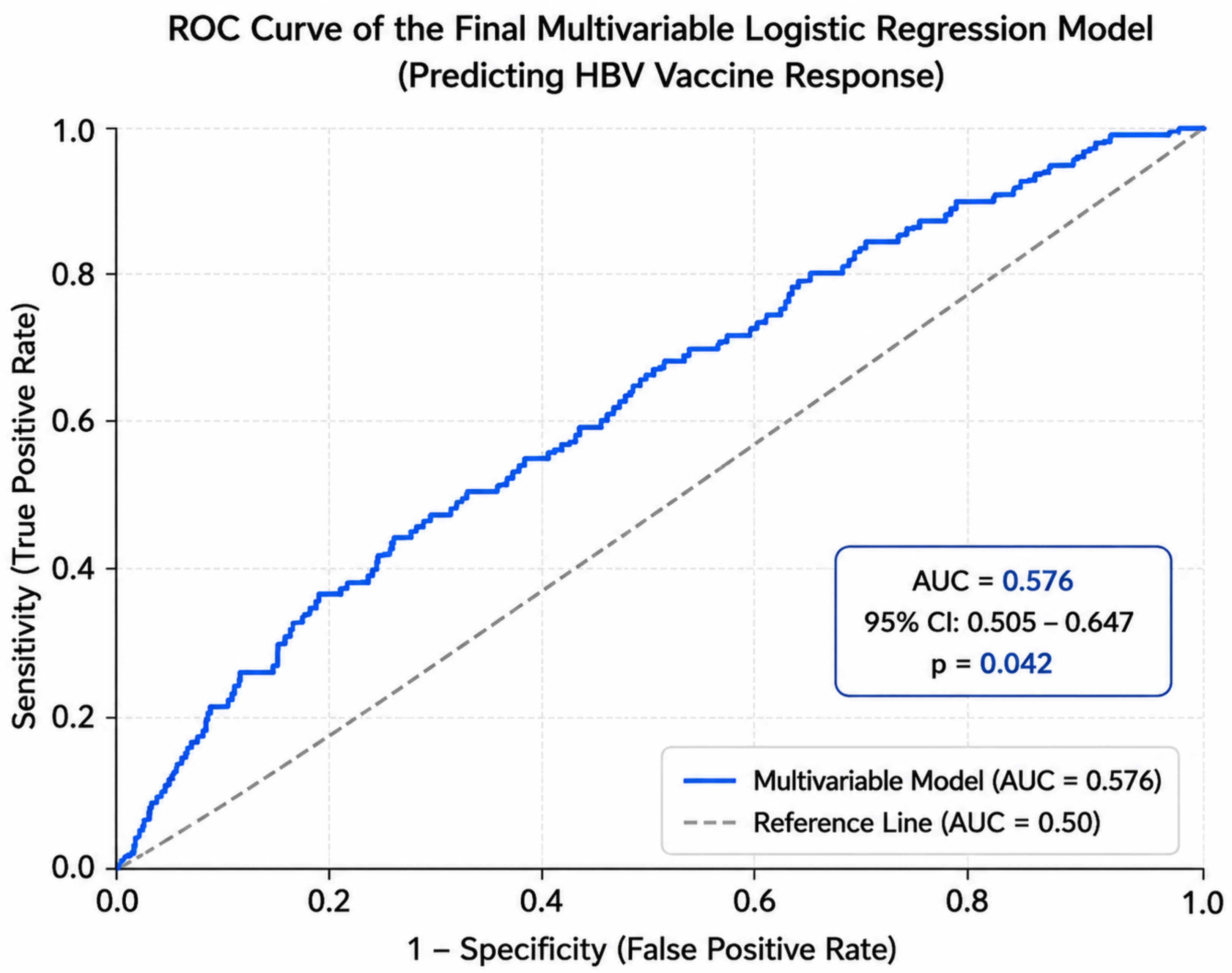

Model adjusted for age, HDL cholesterol, triglycerides, and TG/HDL ratio.
